# A Comparison of Office Policies, with Special Reference to Pulp Exposure or Death

**Published:** 1918-09

**Authors:** Otto E. Inglis

**Affiliations:** Philadelphia, Pa.


### A COMPARISON OF OFFICE POLICIES, WITH SPECIAL REFERENCE TO PULP EXPOSURE OR DEATH

BY OTTO E. INGLIS, D.D.S., PHILADELPHIA, PA.

The dental profession is now face to face with conditions that are to the mind of the writer the outcome of policies definitely pursued by dentists or by their patients with the tacit consent of the dentist. That these policies have been pursued in ignorance, on the part of both dentists and patients, as to the gravity of the outcome, in no wise lessens the difficulties to be faced.

The difficulties lie in the presence in the mouths of a vast majority of people, who have more or less regularly employed dentists—some of them very regularly according to their own accounts—of devitalized teeth numbering from one to eight or ten, with the complications of possible abscesses. Some of these teeth have been well treated mechanically, and perhaps aseptically, or more probably antiseptically, and are without abscesses or granulomas at the apices. Some have been well treated in so far as the root is well filled, or even overfilled, while radiolucent areas are to be found.

A much larger number have been only partially treated, while the canals have been easily susceptible of better filling. In many of these there are no abscesses, in some there are.

It is not the writer's intention to go into the merits of root canal work, but to endeavor to state reasons for there having been a necessity for root work at all. It is obvious that, leaving out consideration of anchorage and bridge work, fixed or removable, in which devitalization was intentional, canal work has been necessitated by pulp exposure or close approach. Why have so many cases of this sort occurred?

There are two answers: (1) The patients have waited too long before consulting the dentist, and have then usually done so because of pain. (2) The dentist has overlooked or neglected cavities existing at the time of examination, and the patient has departed in full confidence of safety, only to suffer a surprise in an attack of pain which again takes him to the dentist.

For the first fault the remedy is education of the *clientèle* before the evil is done; such education as shall convince them that "consultation" early and often is the only method of avoiding serious cavities or pyorrhea alveolaris. The writer has talked this to his patients year in and year out, and is convinced that something more than talk at the chair is necessary to carry conviction even to those who desire to save their teeth, and believes that the time has come for a persistent campaign of education along the indicated line.

Articles should be published at frequent intervals in the daily or other publications, expounding repeatedly the dangers of delay, and pamphlets dealing with various phases of dental disease and with the care of the teeth in general should be distributed to all patients, so that the knowledge shall become widespread in each community. It is by no means intended that these shall take the form of personal advertising, but be authoritative matter issued under the name of local, state or national dental societies without even the name of the writer attached. This will involve expense for the societies in so far as the publication of the pamphlets is concerned, and the societies should be allied in this work, bearing their *pro rata* share according to membership. Outside dentists should also contribute, as it will be equally to their advantage to escape the evils of the ignorance of patients. The pamphlets in condensed form should be published by one or more societies under its or their names, and sold to individual dentists at as near cost as possible for free distribution to the *clientèle* of that dentist, and under ethical agreement. At the present time the public is getting its education largely through personal experience of individuals published in magazines.

In the second case, in which the dentist is at fault, a brief *résumé* of policies employed may be of some benefit.

There is, first, the dentist who, having complete control of his patients, orders them to return at frequent intervals, sufficiently close for at least that form of prophylaxis which keeps cavities to a minimum size, and allows no large ones to form except as an occasional and unavoidable accident. While perhaps not so impeccable as strict monthly prophylaxis, it is still a commendable policy. Whether this dentist employs the policy of strict extension for prevention in all cases of small approximal cavities is a question to be left to individual judgment. The point at issue is that no pulp exposures shall be allowed.

There is, second, the dentist who pursues the policy of filling only cavities which can be extended readily into large contours. A well-known dentist who enjoys a highly paid practice once remarked to the writer that he paid no attention to very small cavities, but saw his patients every three months, filling such cavities as in his judgment required filling. No criticism is made if he comes within the limit of safety, but the writer is convinced that such a policy will in nine cases out of ten lead to pulp exposure, and even worse, to pulp hyperemia and death under well-made fillings. Pyorrhea can, of course, be prevented if the dentist takes pains to observe and treat gingival irritations.

The third class of dentists pursues the policy of attending to such work as is demanded, making little effort to control regular attendance, but such work as is done is well done, and with some effort to complete the work in each mouth. Many exposures are treated, and many pulps die after effort at salvation.

A fifth class keeps the patient in continual attendance, has the office full of patients, places medicated cotton in cavities, and suggests a return next Thursday. Small and usually cash fees are exacted, though in the end the patient pays the price in money, time and destroyed teeth. One dentist of whom the writer knows has thus earned the sobriquet of "Spit and go home," owing to his habit of spending a few minutes on each patient and invariably making that remark. One of his patients is said to have thus spent a year and a half in having one tooth treated, though possibly some slight filling of smaller cavities was done.

The writer would finally enter a plea for frequent prophylaxis, and more especially for thorough cavity diagnosis and filling, for upon the latter much depends. It is not so easy as it seems to diagnose small approximal cavities; even the electric lamp is of no avail in many cases.

In one typical case the writer, upon complaint of the patient, separated two teeth with Perry separators, and found two aproximal cavities with tiny orifices facing and closing each other; then allowed the teeth to resume their positions, and directly afterward, and with full knowledge of their presence, was unable to find them with lamp or explorer.

Even the late Dr. Miller wrote in his textbook that he had seen a number of patients, presumably of excellent dentists, who had consulted him regarding large cavities which he thought had developed during a transatlantic voyage. Without doubt they existed, but were not discovered, before the voyage was begun. Even many occlusal cavities which should be filled are neglected, and frequently the ends of fissures are not extended.

Even with the best intentions and most rigid control of patients, we may be unable to prevent an occasional exposure, but from experience the writer is convinced that such a policy is the only one that can be considered safe and sane at the present day. While it is not orthodox operative dentistry, the writer is firmly of the opinion, based upon twenty years of experience in caring for a considerable number of patients from childhood to adult life, that the strict filling of small approximal cavities as such, conjoined with other prophylactic treatment at short intervals, of say three times a year, is much superior to orthodox contour approximal work carried out because of the presence of larger cavities seen at longer intervals.

It is perfectly true that repairs are at times necessary, and even replacements required, but in the main the teeth are as well saved without pulp exposure, and are of better appearance. As before stated, however, no objection is taken to the preference for extension if, in the judgment of the particular practitioner, this be advisable.—*Cosmos,* August, 1918.

